# Pulmonary sclerosing pneumocytoma containing spindle cells with sarcomatoid features: a case report with literature review

**DOI:** 10.1186/s13000-023-01296-x

**Published:** 2023-02-10

**Authors:** Jing Liang, Qiang Du, Xiaoxing Ye, Wenting Huang

**Affiliations:** 1 Department of Pathology, National Cancer Center/National Clinical Research Center for Cancer/Cancer Hospital, Chinese Academy of Medical Sciences and Peking Union Medical College, Beijing, 100021 China; 2 Department of Pathology, National Cancer Center/National Clinical Research Center for Cancer/Cancer Hospital & Shenzhen Hospital, Chinese Academy of Medical Sciences and Peking Union Medical College, Shenzhen, 518116 China

**Keywords:** Pulmonary sclerosing pneumocytoma, Malignant transformation, Sarcomatoid feature, Immunohistochemistry

## Abstract

**Background:**

Pulmonary sclerosing pneumocytoma (PSP) is an uncommon benign neoplasm originated from pneumocyte and PSP with malignant transformation is extremely rare.

**Case presentation:**

We report a case of PSP of a 65-year-old male patient presented as a lobulated mass in the upper lobe of the left lung, in which part of the stromal round cells transformed to spindle cells with sarcomatoid features and showed no specific differentiation. The patient underwent partial lobectomy without further treatment. No recurrence and metastasis was found after eight month’s follow up.

**Conclusions:**

To our knowledge, this is the first case of PSP with sarcomatoid malignant transformation devoid of differentiation. Our case adds the evidence in that a subset of PSP bear malignant potential and more studies are needed in order to determine the treatment and prognosis to such patients.

## Background

Pulmonary sclerosing pneumocytoma (PSP) is classified as a benign adenoma by World Health Organization classification of tumor and considered to be originated from primitive respiratory epithelium [1]. This tumor is characterized by comprising two types of tumor cells, including surface epithelial cells expressing pan-CK and TTF-1 and stromal round cells expressing TTF-1 and EMA but negative for CK. The morphology of the stromal cells is variant from round, ovoid, polygonal to spindled shape. Sometimes the spindle-shaped stromal cells grow in sheet pattern resembling mesenchymal neoplasms [2]. On the other hand, the biological behavior of PSP is also unpredictable. Although most of the cases of PSP behave in a benign fashion, there are a number of case reports to indicate that a subset of tumors bear malignant potential such as lymph node metastasis or distant metastasis [3–5]. Malignant transformation is extremely rare in PSP [6]. No sarcomatoid transformation of this tumor was described in the literature previously. In this paper, we report a case of a 65-year-old male patient with a PSP in which part of the stromal round cells transformed to sarcomatoid spindle cells without any specific differentiation.

## Case presentation

A 65-year-old male presented in our hospital in December, 2021, who had a lung solid nodular lesion incidentally found by a physical examination 20 days ago. The patient had no specific symptoms to complain and quit smoking for almost 20 years. Chest CT scanning showed a lobulated mass in the upper lobe of the left lung with size of 7.6 × 7.6 × 6 cm. The lesion was well circumscribed under the visceral pleura and no enlarged lymph node was detected. The patient underwent partial lobectomy of the left upper lung in January, 2022. Frozen section examination was performed during operation and a diagnosis of low-grade malignant tumor developed from mesenchymal tissue was made. The patient recovered well after surgery and no recurrence and metastasis was found after eight month’s follow up.

**Fig. 1:**
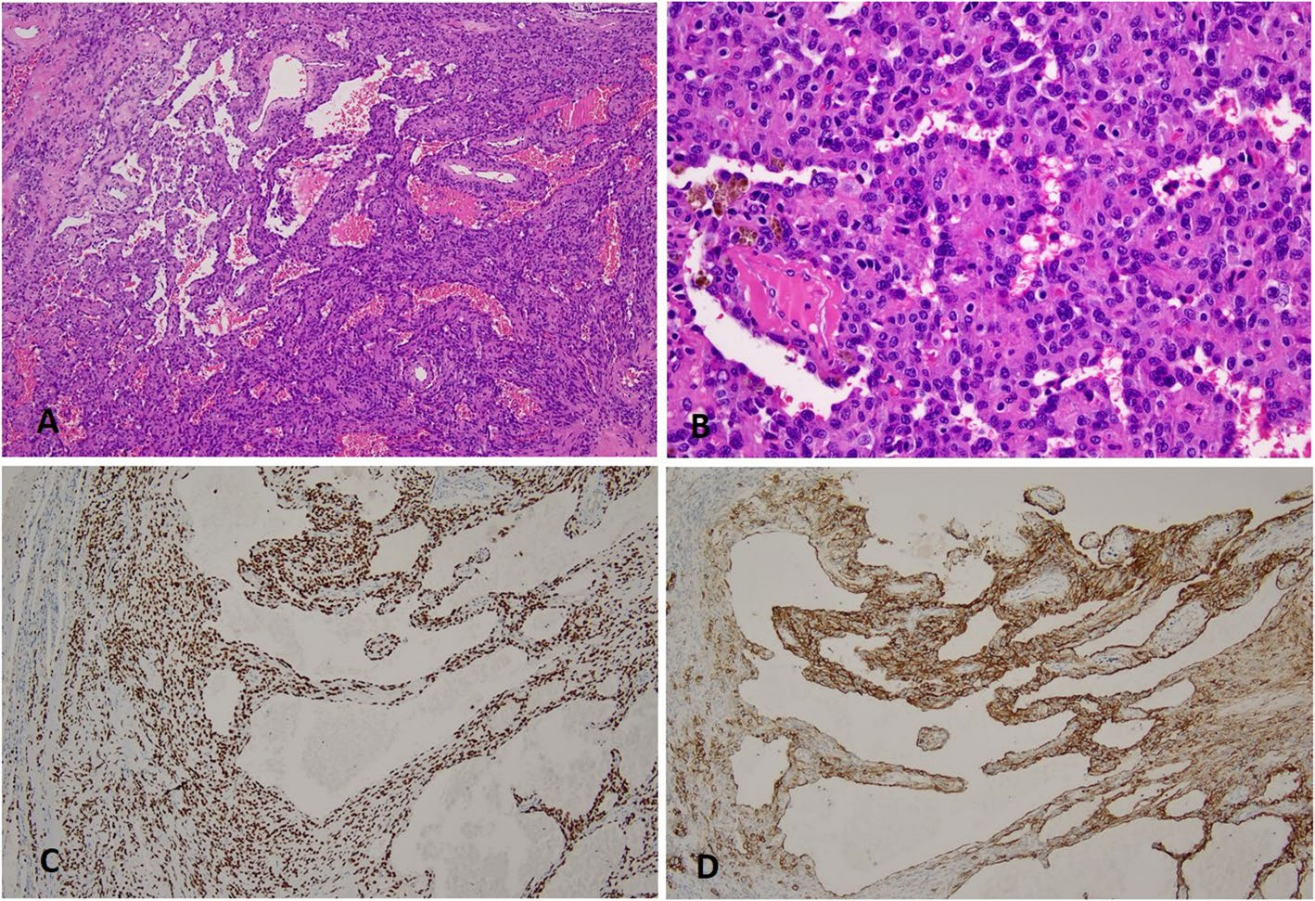
**A**, **B** Typical PSP area of papillary and hemorrhage growth pattern at the periphery of the tumor (H&E stain, × 100, × 200), with positive immunohistochemical staining of TTF-1 (**C**) and EMA (**D**) (× 100)

Gross examination revealed a tumor partly dissociated and partly demarcated sharply with the surrounding pulmonary tissue, with the largest dimension of 8 cm. The tumor was totally solid and showed white–grey on cut surface, with scattered foci of hemorrhage. Microscopically, the tumor consisted of regions with different morphology. At the periphery of the mass, the tumor showed the typical features of pulmonary sclerosing pneumocytoma (Fig. 1). Distinct growth patterns such as papillary, solid and hemangioma-like were seen. Both the lining cuboid cells and the stromal round cells were bland looking and lack of mitotic figure. Immunohistochemical staining confirmed the diagnosis of PSP by positive staining of TTF-1and EMA in both types of cells. CK-pan was positive in only the surface cuboidal cells but not the stromal cells which expressed Vimentin strongly.

**Fig. 2:**
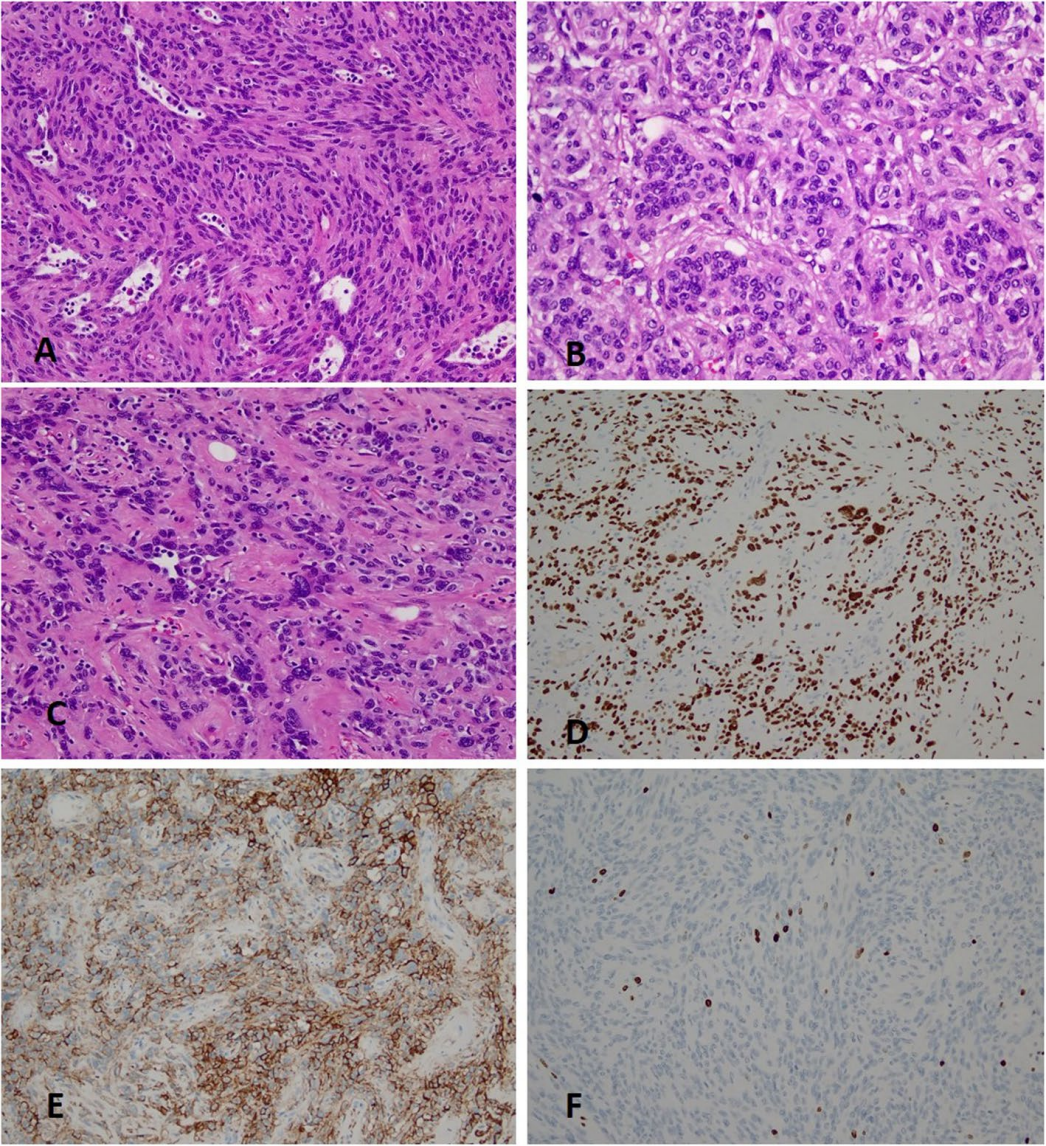
Solid cellular area of the tumor. **A**, **B** The spindle tumor cells are arranged in fascicular and whorled pattern resembling solitary fibrous tumor(H&E stain, × 200); (**C**) In some epithelioid areas, atypical or even bizarre nuclei can be seen (H&E stain, × 200); (**D**,**E**) The atypical cells are positive for TTF-1 and EMA (× 100). (**F**) The Ki-67 index of the spindle tumor cells is low (× 400)

Most areas of the tumor were composed of sheets of compact tumor cells. While some of these cells were small round or polygonal, most of which were oval or spindled with fascicular and whorled growth pattern reminiscent of fibrous or fibroblastic tumors such as solitary fibrous tumor (Fig. 2). In some areas, tumor cells appeared epithelioid with slight to moderate atypical nuclei, and focally bizarre nuclei and multinucleate tumor cells were identified. TTF-1 and EMA were diffusely positive for all these cells with different morphology including the spindle cells and the atypical epithelioid cells, while CK-pan was totally negative, consistent with the phenotype of the stromal cells of PSP. Hence these cellular areas virtually represented enlarged solid pattern of PSP exclusively composed of proliferative and polymorphic stromal round cells. The Ki-67 proliferation index in these areas was low with only slight increase compared to the typical PSP components, with 2–3% and 1%, respectively. Interestingly, the bizarrely shaped nuclei were totally negative for Ki-67, indicating that these cells were not mitotically active.

**Fig. 3:**
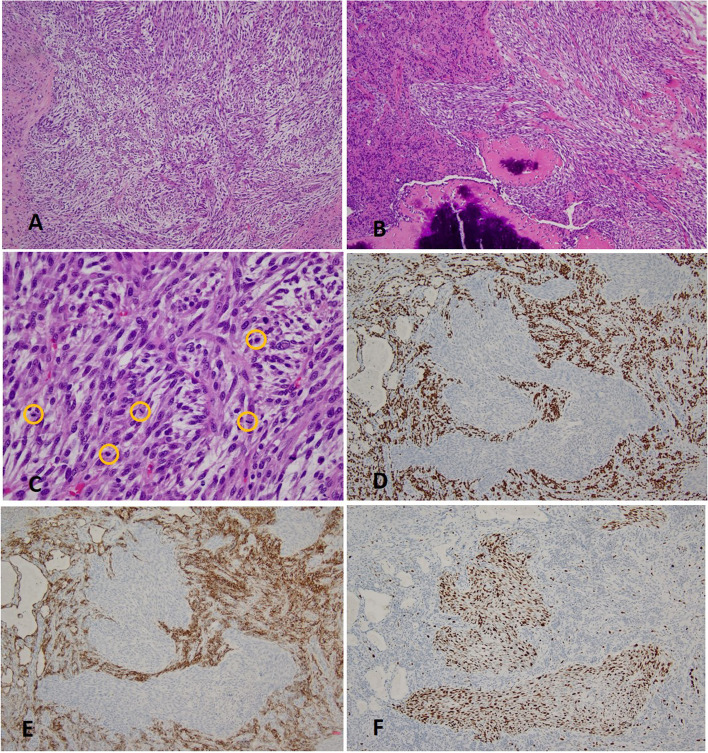
Sarcomatoid area of the tumor. (**A**) The spindle cells arrange in interlacing pattern(H&E stain, × 100); (**B**) The sarcomatoid cells(in the right)intermix with the typical PSP component(in the left) and metaplastic bone can be seen at the bottom of the picture (H&E stain, × 100); (**C**) The monomorphic tumor cells show brisk mitotic activity(yellow circles, H&E stain, × 400); The sarcomatoid spindle cells are negative for TTF-1 (**D**) and EMA (**E**), while the surrounding PSP cells are positive for both markers (× 100); (**F**) Ki-67 index is very high in the sarcomatoid spindle cells (× 100)

In several sections we found some sarcomatoid components amongst the cellular areas. These components were composed of monomorphic spindle cells arranged in long fascicles or storiform patterns, with moderate atypical and hyperchromatic nuclei and relatively prominent nucleoli. The borders of the sarcomatoid areas were irregular and the sarcomatoid cells intersecting with the surrounding tissue were easily seen. In some areas, metaplastic bones were found adjacent to the sarcomatoid spindle cells (Fig. 3). Mitotic activity was fairly high with more than 20 mitotic figures per 10HPF (2mm^2^). For immunostain, the tumor cells were weakly positive for SMA and strongly positive for Vimentin, but negative for other markers such as AE1/AE3, CK7, CK18, CK19, CK5/6, p63, TTF-1, EMA, CD34, desmin and so on. Ki-67 proliferation index was almost 70%.

## Discussion and conclusions

Sclerosing pneumocytoma is a benign tumor of lung comprising two groups of cells, including surface cells resembling type II pneumocytes and round cells located in stroma. This neoplasm was known as sclerosing hemangioma previously and its origin was under controversy for a long time [7–9]. Now it has been accepted that PSP was originated from primary respiratory epithelium and the two groups of cells may come from a common precursor by clonality analysis [1, 10, 11].

Although considered as benign neoplasm, there are dozens of reports revealing the aggressive behavior of this tumor such as bronchial invasion, recurrence, lymph node metastasis and even distant metastasis[3, 4, 12, 13], suggesting that malignant potential may exist in a subset of tumors. Interestingly, despite of the behavior in a malignant fashion, the tumors generally presented as typical PSP morphologically in most of the case reports.Morphological changes in PSP were occasionally described in literature. In a paper summarizing 16 PSP, the lining surface cells of two cases showed atypical hyperplasia [14]. Xu et al. reported a PSP with lymph node metastasis, in which part of the stromal round cells showed pleomorphism and increased nucleoplasmic ratio [3]. In the case report of Teng et al., the malignant transformed stromal cells were shown to have nuclear polymorphism, prominent nucleoli and increased mitotic figures [6]. In rare cases, spindle stromal cells with variant extent were seen in PSP. Gao et al. collected 239 cases of PSP and only 5 cases contained spindle-shaped tumor cells [5]. However, to the best of our knowledge, sarcomatoid spindle cell component was never described in PSP before.

Here we presented the first case of PSP in which stromal cells transforming to spindle cells with sarcomatoid features were found. In this case, most of the tumor was occupied by overgrowth of stromal cells with multiple appearances including ovoid, spindled and epithelioid morphology. Classical PSP components were localized only at the periphery of the lesion, adjoining the overgrowth regions. We considered the later as a putative transitional zone because of the increased cellularity and the relatively higher mitotic activity. Sarcomatoid spindle cells were localized in the transitional regions among the proliferative stromal cells, characterized by uniform histology, fascicular pattern and brisk mitotic activity. The extensive moderate atypia, aggressive growth pattern and high Ki-67 index implied their malignant nature. These cells were positive for Vimentin and SMA but negative for all the other epithelial and mesenchymal markers, suggesting the loss of differentiation in the progression of malignant transformation.

Because of the marked proliferation of spindle stromal cells, there are several mesenchymal tumors that need to be differentiated with our case, including synovial sarcoma, solitary fibrous tumor and, although less possible, a collision tumor in which a sarcoma metastasized to a PSP. The first two entities generally lack the expression of TTF-1. FISH assay was performed and no SS18-SSX gene translocation was found, ruling out the possibility of SS. In addition, immunohistochemical staining showed no expression of CD34 and STAT6 in both the spindled stromal cells and the sarcomatoid cells, not supportive for the diagnosis of SFT. Finally, our patient denied any neoplasm history or surgery history, and imaging examination found no other tumors elsewhere. Hence it was unlikely that this tumor is an admixture of primary PSP and metastatic sarcoma.

Xu et al. summarized 18 cases of PSP with lymph node metastasis and found that the average tumor size with metastasis was bigger than the ones without metastasis(52 mm and 26 mm respectively) [3]. The malignant transformed tumor such as Teng’s and our case were 5 cm [6] and 8 cm in the largest dimension, respectively. It seems that bigger tumor size was associated with the increased malignant potential of PSP. However, the risk factors for malignant potential remains to be determined since malignant transformation is extremely rare in PSP and more data are needed to be collected. It is noteworthy that the spindle cell components, even those metastasized to lymph node kept the expression of TTF-1 and EMA in Teng and other researcher’s cases and were considered as morphologic variant of the stromal round cells [2, 5, 6]. However, the sarcomatoid cells in our case completely lost the expression of these two markers, suggesting a progression of dedifferentiation. Epithelial-mesenchymal transition (EMT) may play an important role in this process and the underlying molecular changes remain to be elucidated.

In summary, we reported the first case of pulmonary sclerosing pneumocytoma containing spindle cells with sarcomatoid features. Our case indicated that the stromal round cells of PSP possess the potential to transform to sarcomatoid spindle cells, and the extensive proliferation of the stromal cells may represent a transition between the classical PSP and sarcomatoid components. Our patient in this case recovered well without recurrence and metastasis eight months after the surgery. However, given the rare number of the malignant transformed cases, the biological behavior of this kind of tumor is still unclear and the prognosis and management to these patients remain to be studied.

## Data Availability

All data generated or analysed during this study are included in this published article.

## Abbreviations

PSP: Pulmonary sclerosing pneumocytoma
EMT: Epithelial-mesenchymal transition
SS: Synovial sarcoma
SFT: Solitary fibrous tumor
